# A probe-treatment-reference (PTR) model for the analysis of oligonucleotide expression microarrays

**DOI:** 10.1186/1471-2105-9-194

**Published:** 2008-04-14

**Authors:** Huanying Ge, Chao Cheng, Lei M Li

**Affiliations:** 1 Molecular and Computational Biology, Department of Biology Sciences, University of Southern California, Los Angeles, CA 90089-2910, USA; 2 Department of Mathematics, University of Southern California, Los Angeles, CA 90089-2910, USA

## Abstract

**Background:**

Microarray pre-processing usually consists of normalization and summarization. Normalization aims to remove non-biological variations across different arrays. The normalization algorithms generally require the specification of reference and target arrays. The issue of reference selection has not been fully addressed. Summarization aims to estimate the transcript abundance from normalized intensities. In this paper, we consider normalization and summarization jointly by a new strategy of reference selection.

**Results:**

We propose a Probe-Treatment-Reference (PTR) model to streamline normalization and summarization by allowing multiple references. We estimate parameters in the model by the Least Absolute Deviations (LAD) approach and implement the computation by median polishing. We show that the LAD estimator is robust in the sense that it has bounded influence in the three-factor PTR model. This model fitting, implicitly, defines an "optimal reference" for each probe-set. We evaluate the effectiveness of the PTR method by two Affymetrix spike-in data sets. Our method reduces the variations of non-differentially expressed genes and thereby increases the detection power of differentially expressed genes.

**Conclusion:**

Our results indicate that the reference effect is important and should be considered in microarray pre-processing. The proposed PTR method is a general framework to deal with the issue of reference selection and can readily be applied to existing normalization algorithms such as the invariant-set, sub-array and quantile method.

## Background

Microarray is one of the most successful techniques in the field of functional genomics. As a high throughput approach, it provides a global gene expression profile of a living cell under certain conditions. The Affymetrix expression microarray is the most widely-used platform. It uses 11–20 probes which have 25 oligonucleotide bases, to represent one gene, and as a whole they are called a probe-set. Associated with each perfect match (PM) probe, a mis-match (MM) probe that differs only in the middle (13^*th*^) base is included in some expression arrays.

From each array, we get fluorescence intensity of each probe after image processing. The estimation of gene expression from probe intensities is a statistical problem where much effort has been made. Among them are Affymetrix's MAS 5.0 [1], Li and Wong's dChip [2-4], and RMA [5-7]. Each method mainly consists of two modules: normalization and summarization. Normalization aims to reduce non-biological variations that are introduced during sample preparation, hybridization and instrumental reading. Summarization combines normalized signal values in each probe-set and produces a final abundance estimate for the corresponding gene.

Normalization requires the specifications of reference and target arrays. We can normalize target arrays against a reference, no matter whether it is a raw microarray or an artificially-defined one. In the invariant-set normalization, which is part of the dChip software, the median intensity of each array is calculated, and the array with the median of these overall medians is chosen as the reference; however, other options are also allowed. The normalization is carried out according to the smooth curve fitted from the rank-invariant intensity set between the reference and target array [2]. The quantile normalization in RMA uses complete data information and defines a pseudo-reference by the averaged quantiles. The normalization is carried out based on the quantile-quantile transformation [5,8]. In the sub-array normalization, once a reference and a target are given, it divides the whole array into sub-arrays and normalizes probe intensities within each sub-array using least trimmed squares (LTS) regression method [9-11]. However, the issue of reference selection has not been well addressed.

In this paper, we propose a Probe-Treatment-Reference (PTR) model that takes into account the reference-effect. The method based on this model aims to streamline normalization and summarization by allowing multiple references. Instead of one reference array, it uses a reference set to do microarray pre-processing. Compared to the two-factor model in [6] and [12], which contains probe- and array-specific effect (later we refer to it as the PA model), we reformulate the array-specific factor by a treatment factor and a reference factor in the PTR model. We estimate the parameters in the model by the least absolute deviations (LAD) approach, which is robust in the sense of bounded influence [13].

The proposed PTR method tries to integrate normalization with summarization in a unified framework. It can be applied to several existing normalization methods, and is particularly useful for the sub-array normalization which aims to reduce spatial variation. Because the reference-specific effect is adjusted at the probe-set level, implicitly, from the reference set, our model defines an "optimal reference" for each probe-set, which may not come from the same raw array. We show that the fold-change estimates from the PTR method are resistant to a bad reference array. It reduces the variation of non-differentially expressed genes and thereby increases the detection power of differentially expressed genes.

## Results

### Microarray data sets

The Affymetrix HG-U133A data set [14] contains triplicates of 14 experiments with 42 spike-in genes. These spike-in genes are organized into 14 gene groups, each of which contains 3 genes of the same concentration. The concentrations range from 0–512 pM according to a 14 × 14 Latin square design. In this paper, we analyze experiment 3 and 4 particularly. Experiment 3 contains the triplicate: *Exp03_R1*, *Exp03_R2 *and *Exp03_R3 *; experiment 4 contains the triplicate: *Exp04_R1*, *Exp04_R2 *and *Exp04_R3*. The concentrations of 36 spike-in genes in experiment 4 are two-fold higher; the concentrations of the remaining 6 spike-in genes are 0 in either experiment 3 or 4. Later we refer to these six arrays as data set "Expt-3-4". Besides, we generate a perturbed data set by adding noise to array *Exp03_R1 *and keeping the other five arrays unchanged. The noise value is defined by *δ*_*i *_= *max*(0, *x*_*i*_), where *x*_*i *_is a normally distributed random variable with a zero mean and variance equal to that of the array *Exp03_R1*. We denote this perturbed array by Exp03_R1*.

We use another data set, the "Golden spike" data set of Choe *et al*. in 2005 [15], to assess the detection power of differentially expressed genes. The data set contains six Affymetrix DrosGenome1 chips, three replicates for control and three for treatment. A total of 1309 genes are differentially expressed with pre-defined fold changes from the set, {1.2, 1.5, 1.7, 2.0, 2.5, 3.0, 3.5, 4.0}, between the treatment and control group.

### The PTR method

**Figure 1:**
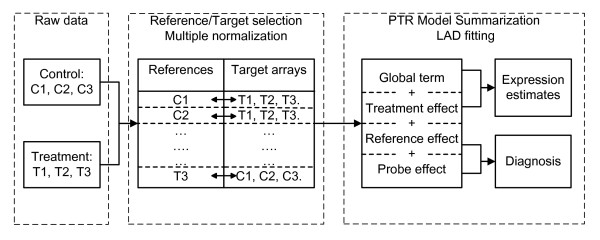
**The scheme of the PTR method**. It includes the reference and target selection, multiple normalization, and three-factor model fitting of summarization. Here, we only illustrate the cross strategy for the reference and target selection.

The PTR method provides an integrative view of normalization and summarization. The scheme is shown in Figure 1. In what follows, we illustrate it by a typical treatment-control case, in which we have three replicates for control and three for treatment. The first step is the selection of references and their target arrays. We propose two simple and straightforward strategies as follows.

• The all-pairwise strategy: all arrays are included in the reference set; for each reference, all the other arrays are its targets.

• The cross strategy: all arrays are included in the reference set; for each reference from control, we take replicates from treatment as its target arrays; conversely, for each reference from treatment, we take replicates from control as its target arrays.

Second, we use existing algorithms such as the invariant-set, sub-array and quantile, to do normalization between each reference and its target arrays. In this paper, we use the function *normalize.AffyBatch.invariantset *in the BioConductor's affy package [16] to carry out the invariant-set normalization. We keep the default parameter settings but allow the reference to be selected manually. We use the procedure described in [9] for the sub-array normalization. The window size is 25, the overlapping size is 10 and the trimming factor is 0.55 for the HG-U133A dataset.

We write a new R function for the quantile normalization. The algorithm is not identically the same as the one [8] used in the RMA package. That is, we normalize each target array against a raw reference array as follows: sort the reference and the target array respectively in the ascending order; replace the sorted intensity values in each target array with those in the reference array; rearrange intensities in each target array according to their original orders [10].

Finally, for each probe-set, we summarize the arrays from log-transformed PM intensities according to the three-factor PTR model:

*log*_2_(Intensity) = global term + treatment effect + probe effect + reference effect + error.

The model states that the variation of logarithmic probe signal values could be sufficiently explained by treatments, probe affinities and references used in normalization. The global term and treatment effects are taken to be the final transcript abundance estimates; the reference effects and the probe effects could be used for diagnosis.

### Perturbed data set

**Figure 2:**
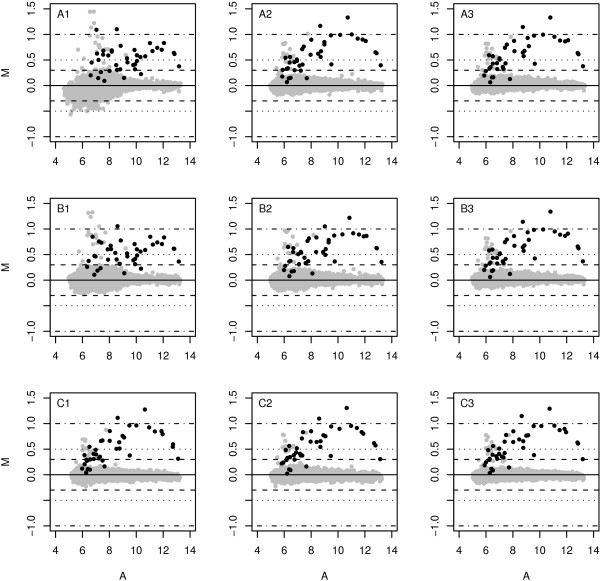
**M-A plots of the perturbed data set using different normalization and reference selections**. Top (A1-A3): invariant-set; middle (B1-B3): quantile; bottom (C1-C3): sub-array. Left column (A1, B1 and C1): the reference is the perturbed array Exp03_R1*; Middle column: the reference in both A2 and C2 is *Exp03_R2*, while the reference in B2 is the pseudo-reference defined as the average quantiles of all six arrays; Right column (A3, B3 and C3): the result obtained by the PTR method using all six arrays as references. The grey dots are non-spike-in genes; the black dots are spike-in genes which are expected to have log-ratio M = 1. We can see that the PTR method results are not affected by the perturbed array Exp03_R1* and offers the smallest variation for non-spike-in genes.

Because the PTR method uses multiple references and the robust LAD estimation, we expect that the expression estimates are resistant to a single bad reference array. In Figure 2, we show the M-A plots of the perturbed data set obtained by different combinations of normalization and reference selections. In these M-A plots, M values of all probe-sets are the log-ratios of experiment 4 versus experiment 3 and A values are the average log-intensities of the two experiments.

The top, middle, and bottom row show the results from the invariant-set, quantile, and sub-array normalization respectively. In A1-B1-C1, we take the perturbed array Exp03_R1* as the reference; in A2-C2, we take the array *Exp03_R2 *as the reference, while in B2, we take the average quantiles as the pseudo-reference; in A3-B3-C3, we simply use the PTR method coupled with the reference set of all six arrays. We see that the invariant-set is more sensitive to the perturbed reference array Exp03_R1* (A1) and it performs well if an appropriate reference is selected (A2). The quantile method shows a similar but less severe phenomenon when the quantile-quantile transformation is based on the array Exp03_R1*. In contrast, the quantile normalization using average quantiles as the pseudo-reference is more resistant to this perturbation. For the sub-array normalization, the perturbation does not affect the ratio estimates, since the normalization transformations are done in each sub-window which greatly reduces the effects of this global noise.

The PTR method improves the performance of all three normalization algorithms. We see that the log-ratios of spike-in genes are closer to the expected log-ratio value *M *= 1, and non-spike-in genes have smaller variations along *M *= 0. The PTR method deals with the single perturbed reference array Exp03_R1* well, and it estimates the final transcript abundance robustly.

### Variation reduction by the PTR method

**Figure 3:**
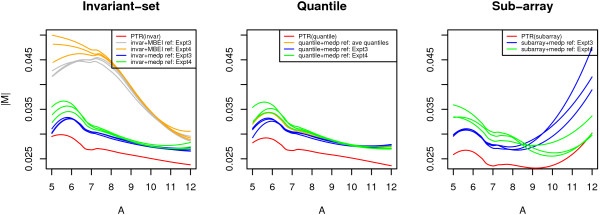
**The LOESS curves of |*M*| versus A by various pre-processing method**. These plots compare the PTR method with other pre-processing methods based on the variation assessment of non-spike-in genes. The PTR method gives the smallest variation for all three normalization algorithms.

It is shown that the PTR method achieves smaller variances for the non-spike-in genes in the M-A plots of the perturbed data set. To further measure the effectiveness of the PTR method in variation reduction, we compare it with other pre-processing methods using "Expt-3-4" data set. The non-spike-in genes are expected to have the same expression in both experiment 3 and 4, and the differences between them are due to the non-biological factors. We pre-process six arrays with different methods of normalization and summarization and estimate the expression values for both experiment 3 and 4. In the M-A plots, we fit LOESS curves [17] to the absolute values of M against A using non-spike-in genes only. The curves shown in Figure 3 measure the variations of non-spike-in genes between two experiments. The left, middle, and right plot respectively show the results from the invariant-set, quantile, and sub-array normalization method. Except for the PTR method, we summarize the normalized results using the *expresso *function in the affy package [16], with either the Li-Wong's MBEI [3] or the RMA's median polish [6] method. From the plots, we see that the PTR method achieves the smallest variations for each normalization algorithm. In the left plot, we use the invariant-set normalization for both the PTR method and the other single-reference methods. The plot shows that the same pre-processing method results in different variation curves with different reference choices. The PTR method reduces the background noise significantly.

In the middle plot, we use the quantile normalization, taking each raw array as well as the average quantiles to be the reference. The plot shows substantial improvement by the PTR method, while the quantile normalization achieves a moderate performance using the average quantiles as the pseudo-reference. The PTR method provides an alternative to use the complete data information.

In the right plot of Figure 3, we use the sub-array normalization, and once again the PTR method improves the performance. Furthermore, the curves from the single reference results can be clustered into two categories. Each reference array from experiment 3 performs better at the low-intensity range but worse in the high-intensity range; whereas each from experiment 4 performs slightly better in the high-intensity range but worse in the low-intensity range. A similar yet less prominent pattern also exists in the results using the invariant-set and quantile normalization. Replicates within either experiment 3 or 4 are similar, but arrays from two experiments lead to more different results. The PTR method takes reference arrays from both experiments, combines two types of expression results and gives the smallest variation in both low- and high-intensity ranges.

The LOESS assessment has demonstrated that the PTR method can reduce the variation significantly for the invariant-set, quantile and sub-array normalization method. This variation reduction, especially in the low-intensity range, is particularly valuable in the measurement of gene expressions.

### Improvement on detection of differentially expressed genes

**Figure 4:**
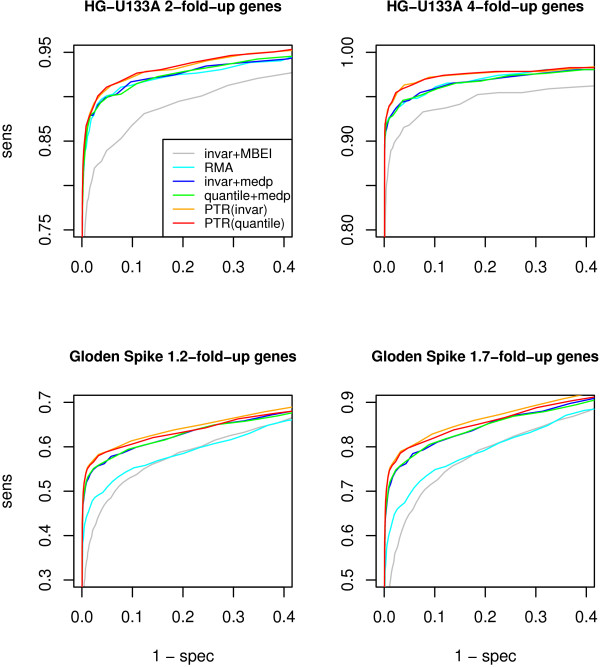
**ROC curves of the PTR and other methods**. X-axis: 1 – specificity; Y-axis: sensitivity. The PTR method performs the best in all cases.

In order to ensure that the detection of differentially expressed genes is not sacrificed at the effort of variation reduction by the PTR method in expression analysis, we compute the receiver operating characteristic (ROC) curves [6] for the HG-U133A data set. We conduct two comparative studies on this data set. In the first study, we compare every two experiments which are next to each other in the Latin square design. Each pair of experiments have 36 spike-in genes with 2-fold change. In the second study, we compare every two experiments which are separated by one and exactly one experiment. Each pair of experiments have 36 spike-in genes with 4-fold change. In total, we have 14 pairs of experiments in each study. The curves computed by the ROC package from Bioconductor [18] are shown at the top of Figure 4. We compare the PTR method with various pre-processing combinations in the *expresso *function of the affy package [16], which includes: invariant-set normalization plus Li-Wong's MBEI or RMA's median polish summarization; quantile normalization plus RMA's median polish summarization with or without RMA's background correction. We see that the PTR method does equally well with both the quantile and invariant-set normalization algorithm in this data set. The PTR method gives the best performance in the detection of the differentially expressed genes.

In addition, we make the similar comparisons on the "golden spike" data set. We set the differentially expressed genes using fold change threshold to 1.2 or 1.7. The ROC curves are shown at the bottom of the Figure 4. We can see that the PTR method performs better with the invariant-set normalization than the quantile normalization. However, both of them outperform the other pre-processing methods.

The assessment on both data sets demonstrates the higher specificity and sensitivity achieved by the PTR method. The noise reduction improves the detection of deferentially expressed genes.

### Implicit reference selection in the PTR model-fitting

**Figure 5:**
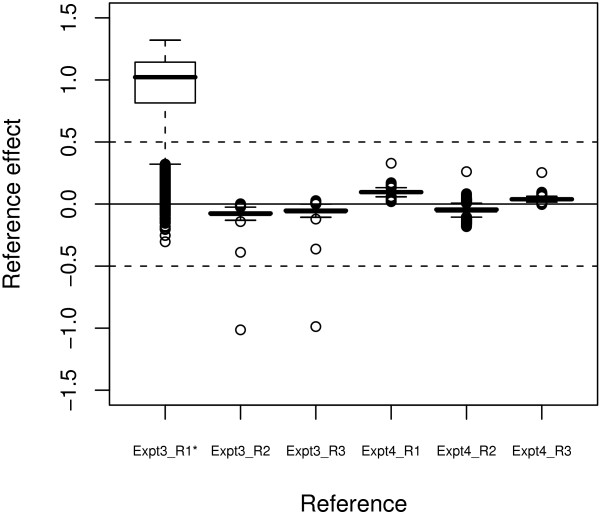
**Distribution of the reference effect**. This reference effect box plot is get from the PTR model-fitting on the perturbed data set after the invariant-set normalization. The first reference array, Exp03_R1*, has been perturbed by adding noise. It shows a quite different distribution than others.

Instead of selecting a single reference, we adjust for the reference effect of each probe-set by the PTR model. The final transcript abundances are obtained from normalized arrays using multiple references. In what follows we look in details how the three-factor model deals with the reference issue. On the one hand, we directly estimate the effect of each reference used in normalization at the probe-set level. It is interesting to consider the estimates of the reference-specific effect of all probe-sets after the summarization. In the box plots of Figure 5, we show the distributions of six reference effects in the perturbed data set. The variations of the reference-specific effect are quite small in the five unperturbed references compared to the perturbed Exp03_R1* reference, which shows an abnormal pattern. This abnormal variation in the reference effect means that the transformation relations based on reference Exp03_R1* are more different across all the probe-sets and indicates its relative worse status.

**Figure 6:**
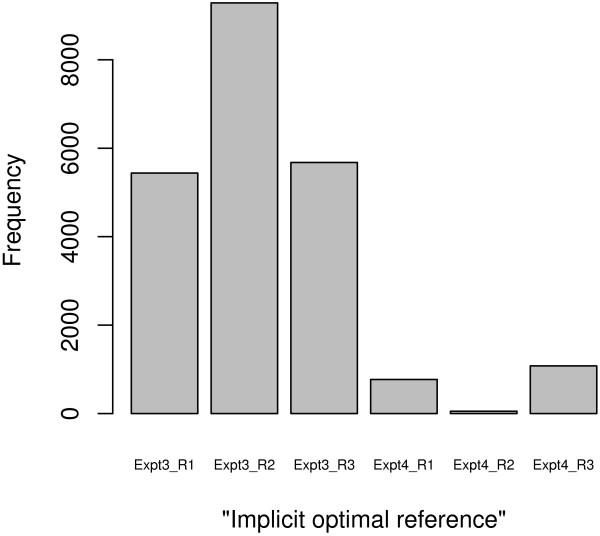
**The frequency of being the "implicit optimal reference"**. It illustrates the frequency of reference arrays which have been served as the "implicit optimal reference" across all the probe-sets. It is computed from the residual assessment after the PTR method with the invariant-set normalization on the data set "Expt-3-4".

On the other hand, the three-factor PTR model provides better fit than the two-factor PA model in term of smaller residual deviations. We can categorize residuals according to their reference indices, and calculate the sum of absolute deviations (SAE, see method) for each residual category. In a sense, the reference that achieves the minimum SAE is the "implicit optimal reference" selected by the model-fitting. In Figure 6, we show the frequency of the "implicit" references across all the probe-sets selected from the pre-processing of the data set "Expt-3-4". The three arrays in experiment 3 are selected more than those in experiment 4. It is consistent with the results from variation (LOESS) curve assessment which demonstrates that each single array from experiment 3 outperforms every single array from experiment 4. The results shown in Figure 5 and 6 are obtained by the PTR method coupled with the invariant-set normalization, and the same phenomenon is also observed using the other normalization methods.

## Discussion

We explicitly address the reference issue in pre-processing expression microarray and propose the PTR method to carry out normalization and summarization jointly. The PTR method can be applied to existing normalization methods such as the invariant-set, sub-array, and quantile. Particularly, it is the first time we propose a practical scheme to implement the sub-array normalization. The sub-array takes into account the spatial pattern of hybridization, and can properly normalize the majority of probe intensities even in the presence of scratches or serious non-homogeneous hybridization. The PTR method enhances this ability using multiple references in normalization and implicitly selecting an "optimal reference" for each probe-set during summarization. In general, the PTR method is applicable to pre-processing biological measurements from array-type instruments such as exon arrays, beads arrays, pathway arrays, tissue arrays and other customized arrays.

The proposed PTR method primarily aims to measure expressions by Affymetrix chips with technical replicates as in many designed microarray experiments. In the normalization step, the sample information is not required although the processing involves multiple pairwise comparisons. In the summarization step, the sample information is necessary for estimating expression differentiation. In some situations, especially the class discovery and class prediction in the cancer study, no technical replicates are available for each sample. To apply the PTR method, we need to assign different treatment labels to different samples that are not replicates in any sense. In this case, one microarray has served as two functional parts: array- and reference-specific effect. For now we could not rule out the possibility of partial confounding of array- and reference-specific effect in general. But we argue that, according to our scheme, normalization is carried out by pooling all probe-sets while summarization is carried out for each probe-set separately.

Consequently, we expect that the reference-specific parameter in each probe-set reflects reference array's block effect in the present of other probe-sets. The association between array- and reference-specific effect should not be significant. It is particular true in the randomized designed Affymetrix chips.

We have not discussed the background correction issue, but the existing background correction methods can be implemented before the normalization step of the PTR method. Interestingly, we have shown that the PTR method reduces the variation of non-differentially expressed genes, especially in the low intensity range. As a consequence, we improve the signal-to-noise ratio and increase the detection power of the deferentially expressed genes. The improvement is achieved without any additional information.

We include all arrays in the reference set and use the cross strategy to select the corresponding target arrays for the data set in this paper. We are aware that they may not be the optimal strategy for reference and target selection. However, the PTR method can protect against and detect abnormal arrays. First, by using the robust LAD method in the summarization, we can accurately estimate the transcript abundance as long as the majority of normalization results are right. Second, we can evaluate the array quality by the reference effect distribution, as shown in Figure 5. The array showing distinct distribution from other arrays is likely to be a bad candidate for reference and may be excluded from the reference set in the next-round PTR processing.

The computational complexity of the PTR method can be decomposed into two parts: normalization and summarization. In an experiment with N arrays, at most we need to carry out N(N-1) pairwise normalization. Thus the complexity is approximately N times of that of single-reference normalization. The median polishing algorithm used in the summarization is an iterative procedure. Compared to the algorithm used in RMA, the median polishing in the PTR is three-way instead of two-way with approximately N times the memory. According to our experience, it does not need substantially more iterations to achieve the same accuracy.

The current implementation of the PTR method needs the R running environment for data input/output and visualization. The normalization part could be carried out by C++ or R depending on the selection of normalization methods. The core computation of the PTR summarization is written in C++ and is called by an R interface. Take the "Expt-3-4" for example, if we use the all-pairwise reference selection strategy, the normalization procedure costs about 2 minutes for the quantile algorithm and 5 minutes for the invariant-set algorithm in a computer with Intel Pentium 2.8 GHz and 4 GB of RAM. The summarization part takes about 10 minutes on the same machine. Due to the specification of memory allocation in R, our current implementation can process a data set up to total 20 arrays in about one hour on the same machine mentioned above. This capability can be expanded if we code the PTR method entirely in C/C++. For a large dataset, we need to adopt algorithms that allocate memory more efficiently. We are now working along this direction to improve the implementation of the PTR method. We also notice that the examples we have examined in the article involve at most three replicates per treatment group. In the case of more samples per treatment group such as 10 or 20, the reference selection may not be a serious issue due to the large sample size. The performance of the PTR method is unclear and the RMA method is a safer choice.

## Conclusion

We propose the Probe-Treatment-Reference (PTR) model to deal with the reference-specific effect. The PTR method streamlines normalization and summarization by allowing multiple references in normalization. It can readily be applied to existing reference-dependent normalization methods. We evaluate the effectiveness of the PTR method on two Affymetrix spike-in data sets. The method reduces the variations of non-differentially expressed genes whereas increases the detection power of the differentially expressed genes.

## Methods

### Probe-treatment-reference model

The scheme of the PTR method is shown in Figure 1. We carry out the PTR model-fitting on each probe-set. The inputs of the fitting are normalized results using multiple references. We note that only PM probes are used in summarization even if the MM probes are available. Let variable *y*_*ijkl *_be the *j*^*th *^normalized probe intensity value of the *l*^*th *^replicate for the *i*^*th *^treatment, with respect to the *k*^*th *^reference array, where *i *= 1, ..., *I*; *j *= 1, ..., *J*; *k *= 1, ..., *K*; *l *= 1, ..., *L*. We postulate that *log*_2_(*y*_*ijkl*_) follows the three-factor model as below:

(1) *log*_2_(*y*_*ijkl*_) = *μ *+ *α*_*i *_+ *β*_*j *_+ *γ*_*k *_+ *ε*_*ijkl*_,

where *μ *is the global term; *α*_*i *_represents the *i*^*th *^treatment-specific effect; *β*_*j *_represents the *j*^*th *^probe-specific effect; and *γ*_*k *_represents the *k*^*th *^reference-specific effect. It is neither simple nor obvious to specify the distribution form for *ε*_*ijkl*_. In addition, they are correlated. In order to make the model identifiable, we impose one of the following constraints:

• Sum constraints: ∑_*i *_*α*_*i *_= 0, ∑_*j *_*β*_*j *_= 0, ∑_*k *_*γ*_*k *_= 0;

• Median constraints: *median*({*α*_*i*_}) = 0, *median*({*β*_*j*_}) = 0, *median*({*γ*_*k*_}) = 0.

We take *μ *+ *α*_*i *_as the final transcript abundance estimate for the *i*^*th *^treatment.

The PTR model is an extension of the RMA's two-factor PA model. Rather than the array-specific effect in the two-factor model, the treatment- and reference-specific effects are used instead in the PTR model. Suppose that we have two treatments and each has three replicates. The two-factor model has 22 (1 + 5 + 10) parameters, assuming the probe-set has 11 PM probes. After the model-fitting, the expression values for each treatment are calculated using the medians or averages across the three replicates. The PTR model has 23 (1 + 1 + 10 + 5) parameters, if we include all six arrays in the reference set. The expression values for each treatment are calculated directly. Meanwhile, it does not necessarily lead to over-fitting if we use more reference arrays, since the number of normalized array increases accordingly. For example, using the cross strategy, three references generate 132 (4 × 11 × 3) normalized PM intensity values, while six references generate 264 (4 × 11 × 6) values. Of course, we are aware that these normalized intensities do correlated to one another.

### Least absolute deviations (LAD) estimation and its computation

To estimate the parameters in the PTR model, we adopt the Least Absolute Deviations (LAD) method. That is, we minimize the sum of absolute errors (SAE):

(2) $$SAE=\sum_{i,j,k,l}\left|\varepsilon_{ijkl}\right|=\sum_{i,j,k,l}\left|log_{2}(y_{ijkl})-\mu -\alpha_{i}-\beta_{j}-\gamma_{k}\right|.$$

The LAD problem can be reformulated as a linear programming (LP) problem and thereby be solved via LP algorithms, such as the simplex and the interior point algorithm [19,20]. We notice that in the PTR model all regressors are categorical variables. It is easily seen that SAE is not minimized if the median of residuals indexed by the same level of a factor is not zero. In this case, the corresponding *α*_*i*_, *β*_*j *_or *γ*_*k *_can be adjusted by making the median zero and this always leads to the reduction of the sum in (2). Therefore, the LAD solution has the property:

$$\{\begin{array}{c} median(\{\varepsilon_{ijkl}\}|i)=0,\\ median(\{\varepsilon_{ijkl}\}|j)=0,\\ median(\{\varepsilon_{ijkl}\}|k)=0. \end{array}$$

Consequently, we can minimize the sum of absolute deviations by the "median polish" approach [21]. Namely, in each step, we sweep out the median for each level of one categorical variable. In each iteration, we run the median polishing through all categorical variables. The SAE is reduced during each step and we stop the iteration once it becomes stable. Our experiences show that at most 20 iterations can give satisfactory results.

### Robustness of LAD estimator

The LAD estimator is the maximum likelihood estimator when the error variable follows a double exponential (Laplace) distribution [13]. Monto Carlo studies also demonstrate that it works well in the cases of a mixture of normals or contaminated normal error distributions [22]. The robustness of LAD can also be quantified by the influence function (IF) which measures the effect of infinitesimal perturbation of one data point on the estimates in the summarization model. We first rewrite the PTR model as follows:

(3) $$Y=X\beta +\varepsilon =\left[\begin{array}{c} x_{1}^{T}\\ \vdots \\ x_{n}^{T} \end{array}\right]\beta +\varepsilon ,$$

where X is the design matrix consisting of -1, 0 and 1; *β *is a vector of parameters including the effects of each probe, treatment and reference; *x*_*i *_is an indicator vector which indexes the signal value *y*_*i *_by its level of each factor. In the example of the "Expt-3-4" data set, we have two treatments and each has three replicates. The probe-set has 11 PM probes, and we include all six arrays in the reference set for the PTR method. Under the sum constraints, *α*_2 _= -*α*_1_; *β*_11 _= *-*(*β*_1 _+ ⋯ + *β*_10_); *γ*_11 _= *-*(*γ*_1 _+ ⋯ + *γ*_5_), and *β*^*T *^= {*μ*, *α*_1_, *β*_1_, ..., *β*_10_, *γ*_1_, ..., *γ*_5_}. The indicator vector,

$$x_{i}^{T}=\{\overset{}{\overset{︷}{\;1\;}},\overset{}{\overset{︷}{\;1\;}},\overset{}{\overset{︷}{0,0,1,0,0,0,0,0,0,0}},\overset{}{\overset{︷}{0,1,0,0,0}}\},$$

means that the signal value *y*_*i *_is from the 1^*st *^treatment, 3^*rd *^probe and 2^*nd *^reference; and the indicator vector,

$$x_{j}^{T}=\{\overset{}{\overset{︷}{\;1\;}},\overset{}{\overset{︷}{-1}},\overset{}{\overset{︷}{-1,-1,\cdots ,-1}},\overset{}{\overset{︷}{-1,\cdots ,-1}}\},$$

means that the signal value *y*_*j *_is from the 2^*nd *^treatment, 11^*th *^probe and 6^*th *^reference.

According to [13,23], the influence function of the LAD estimators at the point (*x*^*T*^, * y*) is given by:

(4) $$\frac{1}{2f(0)}{\left(\frac{1}{n}X^{T}X\right)}^{-1}x\;sign(y-x^{T}\beta ),$$

where *f*(0) is the density function of *ε *at zero and the last term is the sign function, taking value -1, 0, or 1. Since *f*(0) > 0, the influence function of the PTR model is bounded and the largest influence on estimates only depends on *f*(0). We have check the influence of treatment effect estimate in the HG-U133A spike-in data set, and they are less than $\frac{1}{6}$ for most probe-sets.

## Availability and requirements

Software package for the PTR method is available on .

## Authors' contributions

HG wrote the code, carried out the analysis, and drafted the manuscript. CC was involved in analyzing the results and validating the PTR method. LL participated in design and coordination of the study. All 12 authors read and approved the final manuscript.
